# How to Deal With Hemophilia C in Endoscopic Urologic Surgery: Lessons Learned From a Transurethral Resection of Bladder Tumor Case

**DOI:** 10.7759/cureus.36255

**Published:** 2023-03-16

**Authors:** Mehmet Gurcan, Serdar Turan, Ebru Demirel, Senol Tonyali

**Affiliations:** 1 Urology, Istanbul University School of Medicine, Istanbul, TUR; 2 Anesthesiology, Istanbul University School of Medicine, Istanbul, TUR

**Keywords:** factor 11, bleeding times, bladder tumour, hemophilia c, transurethral resection

## Abstract

Factor XI deficiency (hemophilia C or Rosenthal syndrome) is an inherited rare disorder that leads to abnormal bleeding due to the paucity of the protein named factor XI, which plays a role in the blood clotting cascade. A 42-year-old male was referred to the urology outpatient clinic with macroscopic hematuria. The patient was scheduled for a repeat transurethral resection of a bladder tumor (TURBT). Preoperative coagulation parameters were as follows: the international normalized ratio (INR) was 0.95 (0.85-1.2), the prothrombin time was 10.9 seconds (10-15), and the partial thromboplastin time was 43.7 seconds (21-36). On the second postoperative day, he developed pelvic pain and discomfort. An abdominal CT revealed a 10 cm mass consistent with clot retention. The patient received two units of erythrocyte suspension and six units of fresh frozen plasma to prevent the depletion of hemoglobin and control urinary bleeding. The patient was discharged with a good recovery from the hospital three days after the second surgery. Hematologic disorders are rare but might have fatal consequences following surgery if unnoticed at the earliest stage. Clinicians must consider that patients with a history of unusual bleeding or borderline coagulation parameters might have an underlying hematological disorder and perform a further evaluation.

## Introduction

Factor XI deficiency (hemophilia C or Rosenthal syndrome) is an inherited rare disorder with a prevalence of one case per one million people. It leads to abnormal bleeding due to the paucity of the protein named factor XI, which plays a role in the blood clotting cascade [1,2]. This phenomenon can be defined as either partial or severe based on the severity of the deficiency of the factor XI protein. Whereas, regardless of the severity of the protein deficiency, most individuals with this disorder experience relatively mild bleeding problems during their lives. The main characteristic of factor XI deficiency is prolonged bleeding after trauma or surgery, particularly involving the oral cavity, nasal cavity, or urinary tract [3]. Patients with factor XI deficiency may remain asymptomatic until a surgical procedure. If the bleeding occurs after surgery and is left untreated, hematomas can develop in the surgical area.

In this case report, we aim to present a patient who developed a large urinary bladder clot following transurethral resection of a bladder tumor and further treatment. Given the rarity of the situation, it might help to create awareness among urologists about the importance of preoperative coagulation parameters.

## Case presentation

**Figure 1:**
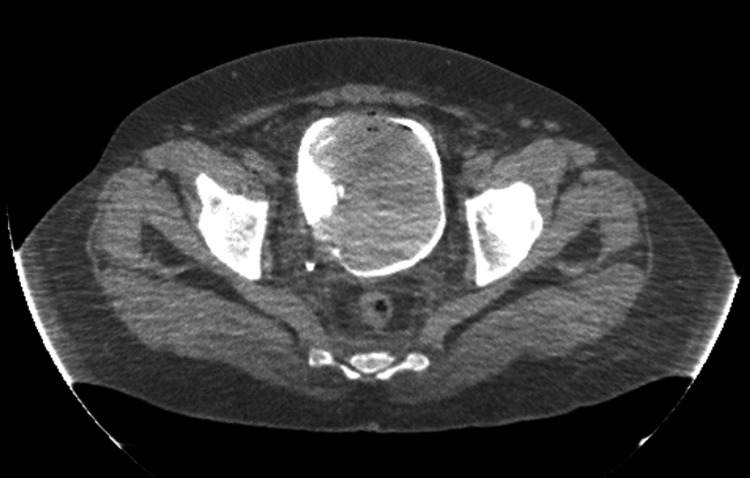
A computed tomography-based cystography showing a 10 cm bladder clot mass

A 42-year-old male was referred to the urology outpatient clinic with macroscopic hematuria. His medical history was without any significant features except a failed transurethral resection of a bladder tumor (TURBT). He was an active smoker for about 25 years. He was not on any medications. On ultrasonographic examination, an 8x6 cm mass suspicious of a bladder tumor was detected in his urinary bladder, and he had undergone an incomplete or failed TURBT in another center that was uneventful. There was no pathological report related to this operation. The patient was scheduled for a repeat TURBT. Preoperative coagulation parameters were as follows: the international normalized ratio (INR) was 0.95 (0.85-1.2), the prothrombin time was 10.9 seconds (10-15), and the partial thromboplastin time was 43.7 seconds (21-36). The hemogram was as follows: hemoglobin: 13.2 g/dl; hematocrit: 40.1%; platelet count: 377,000. Complete removal of the mass by bipolar transurethral resection was performed. The urine color was clear at the end of the operation. However, the patient needed high-volume, continuous catheter saline irrigation due to bleeding in the postoperative recovery room. On the second postoperative day, he developed pelvic pain and discomfort. Manual urinary catheter irrigation with a syringe was also not successful. Bedside ultrasonography was suggestive of a bladder clot. An abdominal computed tomography (CT)-assisted cystography was performed. Abdominal CT revealed a 10 cm mass consistent with clot retention (Figure 1).

The patient underwent surgery for clot removal and extensive coagulation immediately. There was no clear source of bleeding, but there was a tendency for diffuse bleeding in the resected area. Following the operation, we consulted with the hematology department. The patient received two units of erythrocyte suspension and six units of fresh frozen plasma to prevent the depletion of hemoglobin and control urinary bleeding.

Further bleeding tests such as factor VIII, factor IX, factor XI, von Willebrand factor (VWF), and the Ristocetin factor were also performed. The results are as follows: factor VIII: 103.7% (70-150); factor IX: 115.7% (70-120); factor XI: 3.7% (70-120); von Willebrand factor (VWF): 110.7 (70-160); Ristocetin factor: 128.6% (50-150). According to these test results, the patient was diagnosed with factor XI deficiency (Rosenthal disease). The patient was discharged with a good recovery from the hospital three days after the second surgery. The final pathological examination revealed Ta, low-grade urothelial carcinoma. Intracavitary Bacillus Calmette-Guérin immunotherapy was planned. On follow-up, a recurrent bladder tumor was detected, and in the third postoperative month, the patient was scheduled for TURBT for a recurrent bladder tumor. The patient was consulted with the hematology department for preoperative preparation. It was suggested to transfuse 3x2 units of fresh frozen plasma daily starting the day before the surgery until the third postoperative day. When these recommendations were followed, the patient did not experience any bleeding or complications.

## Discussion

Factor XI is primarily synthesized in the liver, and small copies are expressed in renal tubules, pancreatic platelets, and other cell types [4,5]. Factor XI is not effective in the initiation of the coagulation cascade but plays an important role in the continuation of the coagulation cascade. It also acts as both a procoagulant and an antifibrinolytic, playing a role in the regulation of thrombin production.

Patients with factor XI deficiency may remain asymptomatic throughout their lives if they do not undergo a major operation. Spontaneous major bleeding is an extremely rare condition with this deficiency. Bleeding is more common in areas where antifibrinolytic activity is high, such as the oral cavity, pharynx, and genitourinary tract [6]. Our patient also had no history of bleeding, even though he had undergone a TURBT.

Factor XI deficiency should be considered in the differential diagnosis of unpredictable bleeding after any surgery, and platelet count and coagulation parameters should be examined. The activated partial thromboplastin clotting time (APTT) may be normal or borderline high in these patients [7]. In such a case, factor levels should be worked up, including factor XI. Normal levels for factor 11 are 70-150 Iu/l, but there is no correlation between factor levels and bleeding severity. In our case, the factor XI level was extremely low and caused severe bleeding.

Treatment options include fresh frozen plasma, factor XI concentration (only in European countries), desmopressin, fibrin glue, and low-dose recombinant activated coagulation factor V (FVa). Antifibrinolytics such as tranexamic acid can be used for minor bleeding and perioperatively. The most common treatment method is fresh frozen plasma, which we also applied to our patient. Factor XI's half-life is approximately 50-70 hours; therefore, fresh frozen plasma replacement should be performed every 48-72 hours [8,9]. In our case, we used erythrocyte suspensions and fresh frozen plasma and extensively coagulated the resected area of the urinary bladder.

Another issue that must be discussed in this case is the coagulation capacity of the bipolar energy. There is a scarce amount of clinical data comparing the effectiveness of monopolar versus bipolar energy in the transurethral rejection of bladder tumors. Some researchers found bipolar energy superior to monopolar energy in terms of hemoglobin fall following surgery [10].

## Conclusions

Hematologic disorders are rare but might have fatal consequences following surgery if unnoticed at the earliest stage. Preoperative evaluation, including coagulation parameters, is of importance in all patients undergoing surgery. Clinicians must consider that patients with a history of unusual bleeding or borderline coagulation parameters might have an underlying hematological disorder and perform a further evaluation.
